# Identification of miRNAs and Their Targets Involved in Flower and Fruit Development across Domesticated and Wild *Capsicum* Species

**DOI:** 10.3390/ijms22094866

**Published:** 2021-05-04

**Authors:** Carlos Lopez-Ortiz, Yadira Peña-Garcia, Menuka Bhandari, Venkata Lakshmi Abburi, Purushothaman Natarajan, John Stommel, Padma Nimmakayala, Umesh K. Reddy

**Affiliations:** 1 Department of Biology, Gus R. Douglass Institute, West Virginia State University, West Virginia, WV 25112, USA; carlos.ortiz@wvstateu.edu (C.L.-O.); ypenagarcia@wvstateu.edu (Y.P.-G.); mbhandari@wvstateu.edu (M.B.); vabburi@wvstateu.edu (V.L.A.); pnatarajan@wvstateu.edu (P.N.); padma@wvstateu.edu (P.N.); 2 Genetic Improvement of Fruits and Vegetables Laboratory, USDA, ARS, Beltsville, MD 20705, USA; john.stommel@usda.gov

**Keywords:** miRNA, flower, fruit development, *Capsicum* species

## Abstract

MicroRNAs (miRNAs) are regulators of the post-transcription stage of gene activity documented to play central roles in flower and fruit development in model plant species. However, little is known about their roles and differences in domesticated and wild *Capsicum* species. In this study, we used high-throughput sequencing to analyze the miRNA content at three developmental stages (flower, small fruit, and middle fruit) from two cultivated (*C. baccatum* and *C. annuum*) and two wild (*C. chacoense* and *C. eximium*) pepper species. This analysis revealed 22 known and 27 novel miRNAs differentially expressed across species and tissues. A number of stage- and species-specific miRNAs were identified, and Gene Ontology terms were assigned to 138 genes targeted by the miRNAs. Most Gene Ontology terms were for the categories “genetic information processing”, “signaling and cellular processes”, “amino acid metabolism”, and “carbohydrate metabolism”. Enriched KEGG analysis revealed the pathways amino acids, sugar and nucleotide metabolism, starch and sucrose metabolism, and fructose-mannose metabolism among the principal ones regulated by miRNAs during pepper fruit ripening. We predicted miRNA–target gene interactions regulating flowering time and fruit development, including miR156/157 with *SPL* genes, miR159 with GaMYB proteins, miR160 with ARF genes, miR172 with AP2-like transcription factors, and miR408 with *CLAVATA1* gene across the different *Capsicum* species. In addition, novel miRNAs play an important role in regulating interactions potentially controlling plant pathogen defense and fruit quality via fructokinase, alpha-L-arabinofuranosidase, and aromatic and neutral amino acid transporter. Overall, the small RNA-sequencing results from this study represent valuable information that provides a solid foundation for uncovering the miRNA-mediated mechanisms of flower and fruit development between domesticated and wild *Capsicum* species.

## 1. Introduction

MicroRNAs (miRNAs) are a specific class of 20- to 24-nt endogenous small non-protein coding RNAs involved in post-transcriptional and translational gene expression regulation in plants and animals [1,2]. Mature miRNA, generated from longer pri-RNA via nuclease cleavage processes [3], negatively regulate gene expression by recognition and complementary binding to the open reading frame or untranslated regions (UTRs) of target genes. The expression of multiple genes can be regulated by a single miRNA, and multiple miRNAs can control a single gene expression [4]. In plants, the complementarity between miRNA and their targets is very high, which results in RNA-induced silencing complexes by degrading the target mRNA or inhibiting mRNA translation [5]. Therefore, miRNA-mediated gene silencing plays an important role in several essential plant biological processes, including developmental control [6,7], hormone secretion [8], cell differentiation and proliferation [6], as well as environmental adaptation and response to conditions such as salinity, drought, and low temperature [9,10,11,12]. Similarly, some miRNAs mediate plant–microbe associations, which suggests their participation in processes such as symbiosis events and plant–pest interactions [13,14,15]. Nevertheless, the regulation of miRNAs is known to be spatiotemporally specific, so understanding their regulatory roles in plants is difficult [16].

The rapid advances in bioinformatics and next-generation sequencing technologies have led to the continuously increasing identification of novel miRNAs in plants. Different databases provide relevant information on miRNAs; one is miRbase (v22.1, http://www.mirbase.org, accessed on 1 August 2020), which contains about 38,600 entries representing hairpin precursor miRNAs that express 48,860 mature miRNA products in 271 species [17]: 10,414 belong to 82 different plant species, including the model *Arabidopsis thaliana*, and crops such as *Oryza sativa*, *Glycine max*, and *Medicago truncatula*. However, the miRNA annotation of other important plants such as those in the Solanaceae family, with more than 3,000 species [18], remains limited. Currently, miRbase contains miRNA information for only a few members of this family—*Nicotiana tabacum* (164), *Solanum lycopersicum* (147), and *Solanum tuberosum* (343). Pepper (*Capsicum* spp.), which also belongs to the Solanaceae family, exhibits wide diversity, with more than 200 species that vary by color, size, shape, and chemical composition. It is one of the most economically important crops cultivated worldwide because of its economic importance and also its medicinal and nutrimental value. In addition to their dietary and culinary importance, capsaicinoid compounds (capsaicin and dihydrocapsaicin) in pepper have a beneficial effect for humans, including antioxidant, anticarcinogenic, antimutagenic, antiaging, and antibacterial properties [19,20,21].

The release of the pepper (*Capsicum annuum*) genome sequence [19,20] has offered an opportunity to better understand different molecular mechanisms at a transcriptional level. To date, only a few studies have focused on pepper miRNA profiles in *C. annuum* [22,23]; however, considering the importance of pepper, we need to understand the function and expression of miRNAs across different *Capsicum* species. Further information on their expression across the diverse developmental stages may be useful to elucidate the mechanisms involved in gene regulation and can provide insights into the biological processes underlying the environmental adaptation of *Capsicum* species. In this study, we used high-throughput sequencing to identify pepper miRNAs in two cultivated *Capsicum* species, *C. baccatum* and *C. annuum*, and two wild *Capsicum* species, *C. chacoense* and *C. eximium*, at three different developmental stages (flower, small fruit, and middle fruit). Simultaneously, we investigated the dynamic regulation and studied the evolutionary changes of the identified miRNA genes. The identification of putatively conserved and novel miRNAs in inter/intra-species levels across different members of the *Capsicum* genus provides valuable insights into the evolution of the microRNAome with respect to domestication and selection events related to fruit development.

## 2. Results

### 2.1. Analysis of Small RNAs in Pepper

**Figure 1:**
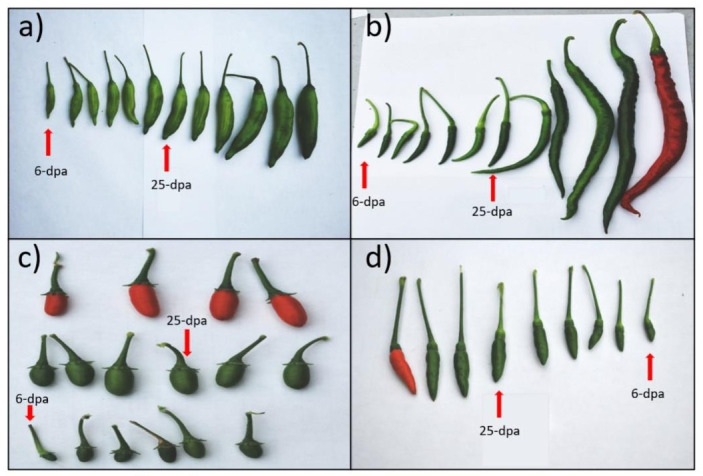
Morphological features of pepper fruit development stages of four *Capsicum* species. Harvested fruit at different developmental stages from days after pollination of (**a**) *C. baccatum*, (**b**) *C. annuum*, (**c**) *C. chacoense*, and (**d**) *C. eximium*. Red arrows indicate small fruit (6-dpa) and middle fruit (25-dpa) stages that were collected for small RNA sequencing.

**Figure 2:**
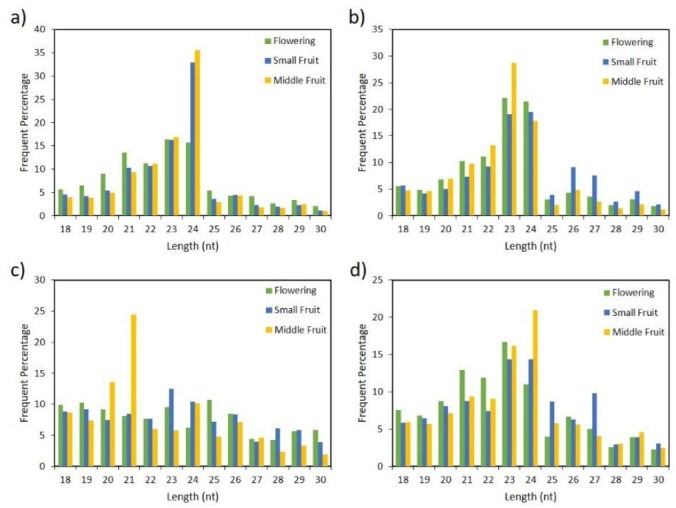
Length distribution of small RNAs in (**a**) *C. baccatum*, (**b**) *C. annuum*, (**c**) *C. chacoense*, and (**d**) *C. eximium* at flowering, small, and medium fruit stages.

For comprehensive analysis of the *Capsicum* miRNAome and to identify putative miRNAs associated with fruit development, we sequenced 12 small-RNA libraries derived from flower, small fruit (6 days post anthesis) and medium fruit (25 days post anthesis) developmental stages of four *Capsicum* species (Figure 1) by using the Illumina HiSeq 2500 instrument. A total of 162,841,574 raw reads were obtained from the 12 libraries. After removing adaptors, low-quality reads, poly-A sequences, and reads of <18 and >30 nt in length, the number of clean reads ranged from ~3 million in *C. annuum* small fruit, to ~8 million in *C. baccatum* small fruit (Table S1). The small RNA clean reads were further classified into different categories by BLAST searches against Rfam and Repbase databases, and noncoding RNAs including rRNA, tRNA, snoRNA, snRNA sequences were removed. The remaining sequences were then mapped to the pepper genome to determine whether they could be candidate miRNAs and were selected based on strict criteria for annotation of plant miRNA. Furthermore, parameters such as length distribution and 5′-end of candidate miRNAs were considered by using the clean reads from all species and tissues (Figure 2). Most of these small RNAs had a 5´-end terminal U or A, which indicates canonical small RNAs [24]. Likewise, the length distribution ranged from 20 to 24 nt, the typical length of canonical miRNAs. Although the size distribution of all small RNAs was diverse and varied across species and tissues, the dominant length was 24 nt followed by 23, 22, and 21 nt in *C. baccatum* and *C. eximium* (Figure 2a,d). However, this length pattern was not observed in *C. chacoense* and *C. annuum* species, whose predominant lengths were 23 nt followed by 24 nt in *C. annuum*; 21 nt was the predominant length in *C. chacoense* at middle fruit stage.

### 2.2. Identification of Known and Novel miRNAs in Pepper

**Figure 3:**
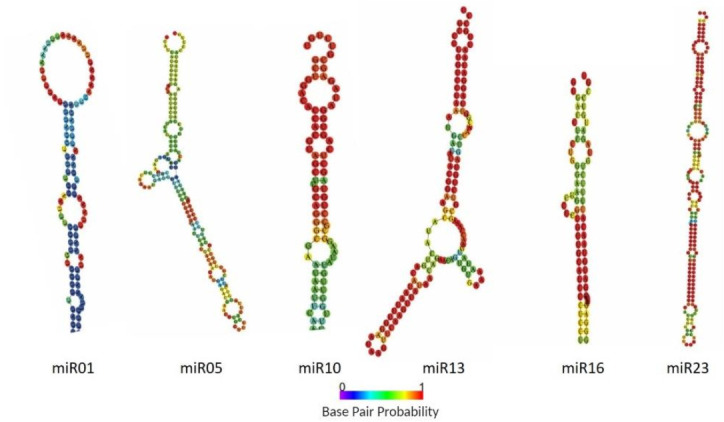
Predicted secondary hairpin structures of six novel miRNAs identified in *Capsicum* species. Base pair probability is represented by color: red, high-probability, and purple, low-probability.

To identify conserved miRNAs in each library, unique sRNA reads were BLASTN searched against currently known mature miRNAs in miRBase (v22.1) allowing one or two mismatches between sequences. Consequently, 22 known miRNAs from 21 conserved families were identified (Table 1). The only miRNA family that showed variants was miR166, with two members (miR166a and miR166b). To predict novel miRNAs in *Capsicum* species, sRNA reads that were unmatched to the miRbase were aligned with the genome sequences of *C. annuum* by using miRDeep2. Ultimately, 27 novel miRNA candidates with stable hairpin structures were identified and designated miR01 to miR27. In addition, precursor sequences from the pepper genome database were obtained (Table S2). Notably, candidate precursors for all the 27 predicted novel miRNAs were suggested. Furthermore, the hairpin structures of these novel small RNAs were predicted by using the RNAfold web server. All the novel miRNAs precursors in pepper possessed typical stem-loop structures and had negative folding free energies ranging from -62.6 to -15.9 according to RNAfold. The predicted secondary structures of six randomly selected novel miRNAs candidates are in Figure 3. In addition, we determined whether these novel miRNA candidates contained miRNA* sequences in the 5p or 3p terminus, which resulted in the identification of the miRNA* sequence in most of the candidate miRNAs, with exception of 6 (Table S2). The first cleavage position is critical to determine the mature miRNA sequence and resulting target specificity [25,26]. Therefore, base composition of miRNAs plays another important role because it may affect secondary structures and biological properties. Nucleotide bias analysis revealed that 21- to 22-nt miRNAs more frequently contained G and U at the first position; however, 23- and 24-nt miRNAs had a strong preference for G across tissues and species (Figure S1). Although the nucleotide bias at each position had wide fluctuation, they followed similar patterns through all species and tissues. This was especially observed at the 10th nucleotide, where the predominant base was G followed by C and U, and at the 11th nucleotide, where the four bases were present with dominancy of base C (Figure S2). Previous studies suggested that the first nucleotide is important for miRNA sorting [26] and that the 10th and 11th nucleotides are responsible for guiding the miRNA to cleave the target mRNA [27]. Altogether, these results suggest that novel miRNAs might be involved in regulating similar physiological and biological processes across *Capsicum* species.

### 2.3. Expression Pattern of Known and Novel miRNAs in Pepper

**Figure 4:**
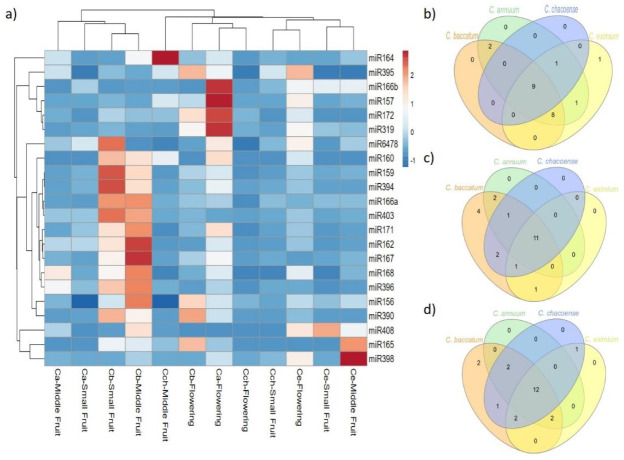
Differential expression analysis of known miRNAs. (**a**) Heat map of known miRNA expression profiles in *C. baccatum* (Cb), *C. annuum* (Ca), *C. chacoense* (Cch), and *C. eximium* (Ce) at different stages analyzed. The expression levels are represented by the color: red, high-expressed; and blue, low-expressed. Venn diagram analysis of shared known miRNAs at (**b**) flowering, (**c**) small, and (**d**) medium fruit stages across *Capsicum* species.

**Figure 5:**
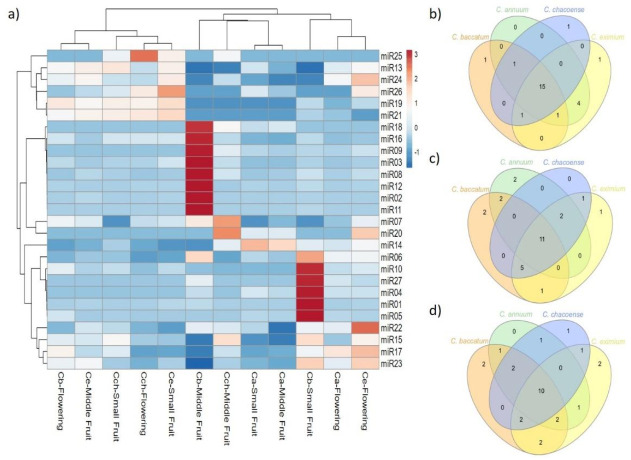
Differential expression analysis of novel miRNAs. (**a**) Heat map of novel miRNA expression profiles in *C. baccatum* (Cb), *C. annuum* (Ca), *C. chacoense* (Cch), and *C. eximium* (Ce) at different stages analyzed. The expression levels are represented by the color: red, high-expressed; and blue, low-expressed. Venn diagram analysis of shared novel miRNAs at (**b**) flowering, (**c**) small, and (**d**) medium fruit stages across *Capsicum* species.

We analyzed the sequence frequencies of known and novel miRNAs from the 12 libraries to estimate the pattern expression of the miRNAs found at a specific developmental stage or tissue and to infer their possible roles in the different *Capsicum* species. Seven of 22 known miRNAs were expressed at different densities in all stages and species (Table 1 and Figure 4): 9, 11 and 12 known miRNAs were expressed at flower, small fruit, and medium fruit, respectively, among all *Capsicum* species. From the miRNA expression at flower stage, *C. baccatum*, *C. annuum*, and *C. chacoense* were clustered together; however, miR157, 166b, 172 and miR319 showed higher expression in *C. annuum*. Furthermore, the expression of 13 miRNAs (miR156, 159, 160, 162, 166a, 167, 168, 171, 390, 394, 396, 403, and 6478) at small and middle fruit stages was higher in *C. baccatum* than the other species. Similarly, miR164, 165 and 398 showed high expression in *C. chacoense* and *C. eximium* at middle fruit stage, whereas miR408 was highly expressed in *C. eximium* at small fruit stage. For novel miRNAs, 8 of 27 were expressed in all tissues and species at different levels (Table 2, Figure 5). Additionally, 15, 11, and 10 were shared between *Capsicum* species at flower, small fruit, and middle fruit stage, respectively (Figure 5b,c,d). Similar to known miRNAs, some novel miRNAs also showed species-specific expression; for instance, 14 miRNAs including miR01, 02, 03, 04, 05, 06, 08, 09, 10, 11, 12, 16, 18, and 27 were upregulated in *C. baccatum* at small and middle fruit stages. Likewise, at the same stages, miR014 showed high expression in *C. annuum*, whereas miR07 and 20 were highly expressed in *C. chacoense*. Moreover, miR17, 22, 23, and 24 showed high abundance at flower stage in *C. eximium*, but miR25 was highly expressed at the same stage in *C. chacoense*. Finally, miR13, 15, 19, and 21 showed low expression patterns across all species and stages.

### 2.4. Prediction of Putative Targets for Known and Novel miRNAs in Pepper

To better understand the function of the identified miRNAs and assuming that plant miRNAs have a nearly perfect match to their target mRNAs, putative target genes were predicted by using the psRNA target program. Additionally, we performed ‘microRNA:mRNA’ seed sequence similarity analysis between known and novel miRNAs and their target mRNAs because the seed sequence is essential for binding to take place (Mullany et al. 2016) (Table S3). We identified 78 and 60 potential candidate targets for known and novel miRNAs, respectively (Table S4). The number of putative target genes for a single miRNA ranged from 1 to 6 for known miRNAs and from 1 to 4 for novel miRNAs. The set of predicted target proteins was functionally characterized by using the BLASTKOALA sequence similarity tool. BLASTKOALA annotated 54 (39.1%) of 138 proteins identified. A group of 21 genes was thought to be involved in genetic information processing, but other genes were predicted to participate in processes such as signaling and cellular processes (11), metabolism (7), environmental information processing (3), amino acid metabolism (2), energy metabolism (2), xenobiotics biodegradation and metabolism (2), carbohydrate metabolism (2), lipid metabolism (1), metabolism of terpenoids and polyketides (1), metabolism of cofactors and vitamins (1), and glycan biosynthesis and metabolism (1) (Figure S3).

Moreover, to gain insights into a global overview of the regulatory functions of the miRNAs across *Capsicum* species, we analyzed the GO terms for 116 of the 138 identified targets. GO analysis suggested the putative participation of miRNA targets in multiple biological processes, molecular functions, and cellular component (Figure S4). The major biological processes predicted for these GO-defined target genes were regulation of transcription, DNA-replication (20), oxidation-reduction process (10), protein phosphorylation (10), auxin-activated signaling pathway (5), and hormone-mediated signaling pathway (4). The molecular functions were mostly classified as DNA binding (25), ATP binding (20), protein serine/threonine kinase activity (13), DNA-binding transcription factor (TF) activity (10), and zinc ion binding (8). For cellular components, most genes were related to nucleus (35), membrane (35), plasma membrane (17), cytoplasm (8), and cytosol (8). Furthermore, enriched KEGG pathway analysis of target genes of differentially expressed miRNAs revealed that miRNAs regulate genes involved in amino sugar and nucleotide sugar metabolism, starch and sucrose metabolism, and fructose-mannose metabolism pathways (Figure S5).

### 2.5. Expression Profile of miRNA Target Genes in C. annuum

**Figure 6:**
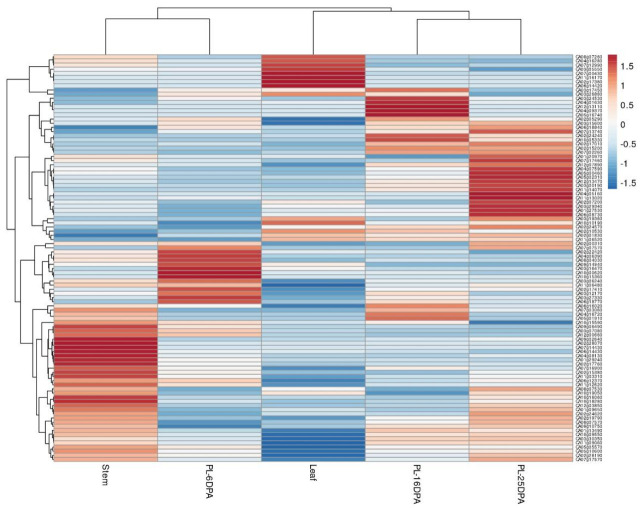
Expression patterns of known and novel miRNA target genes in leaf, stem, and placenta (PL) tissue at 6, 16, and 25 days post-anthesis of *C. annuum* var CM344. The expression levels are represented by color: red, upregulated; and blue, downregulated.

To investigate the expression profile of miRNA predicted target genes across different tissues, including leaf, steam, and placenta tissue at 6, 16, and 25 dpa, we used publicly available RNA-seq data for *C. annuum* cv. CM33428. A heat map was used to visualize tissue-specific expression patterns of target genes (Figure 6). Overall, 112 of 138 predicted genes were identified, and most genes exhibited unique expression profiles. Genes showing exclusively high expression in leaf tissue encoded proteins for *ABC* transporter (CA06g14420), cellulose synthase (CA10g10190), FGGY carbohydrate kinase (CA07g00430), or class III HD-Zip protein *3* (CA02g10530) or were involved in the *PHO* system (CA02g17380). Placenta tissue at 6-dpa showed high expression of genes involved in regulation of transcription such as *SPL* (squamosa promoter binding protein) domain class TF (CA03g12170), *TCP* TF (CA08g04030), and *NAC* (NAM, ATAF, and CUC) domain TF (CA06g18770), thus indicating the participation of these genes in fruit development. The same tissue at 16-dpa showed high expression of genes associated with plant hormone signaling: auxin response factor (CA04g09370) and serine/threonine-protein kinase (CA04g16720). Likewise, genes exhibiting the same profile encoded proteins such as class III HD-Zip protein (CA12g13110), protein SUPPRESSOR OF GENE SILENCING 3 (CA03g17450), and fructokinase activity (CA02g24240). Meanwhile, genes upregulated in placenta tissue at 25 dpa were related to carbohydrate metabolism (CA01g27530, CA03g19360, and CA03g29340), aromatic and neutral amino acid transporter (CA04g05160), leucine rich repeat receptor protein kinase *CLAVATA1* (CA02g24570), and TFs with key roles in ripening such as *AP2* (*APETALA2*) and *NAC* TFs (CA11g14070, CA12g13470). Genes showing high expression during pepper fruit development and ripening at 16 and 25-dpa were the *SPL* TF (CA02g15200) and the response regulator *ARR12-like* protein (CA07g02260).

### 2.6. Validation of miRNA and Target Gene Expression

**Figure 7:**
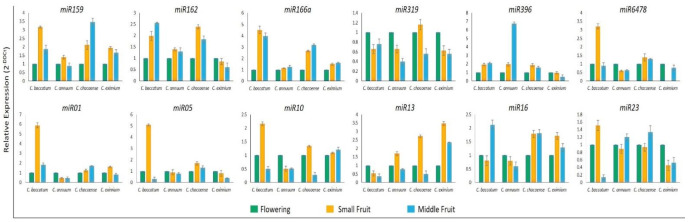
Validation of six known and six novel miRNAs by stem-loop RT-qPCR in *Capsicum* species at flowering, small fruit, and middle fruit stages. The small nuclear RNA (snRNA) U6 was a housekeeping gene. Data are mean ± SD from three biological replicates.

**Figure 8:**
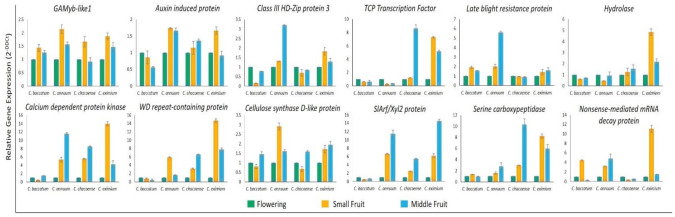
Expression of target genes of known and novel miRNAs in *Capsicum* species at flowering, small fruit, and middle fruit stages. *B-tub* gene was a housekeeping gene. Data are mean ± SD from three biological replicates.

To validate the expression profiles of miRNAs in *Capsicum* species at flower, small fruit (6-dpa), and middle fruit (25-dpa) stages, we investigated 6 known (miR159, 162, 166a, 319, 396, and 6478) and six novel miRNAs (miR01, 05, 10, 13, 16, and 23) by using stem-loop qRT-PCR based on their sequencing frequencies (Figure 7). In general, expression of most of these miRNAs agreed with sequencing data, with slight variation. The expression profiles of miR162, 166a, 319, 396, 6478, 01, 05, and 16 were consistent with the results from sRNA-sequencing. However, the remaining four miRNAs tested showed inconsistent results between stem-loop qRT-PCR and sRNA sequencing in all or specific *Capsicum* species. For instance, miR159 showed a discrepancy in *C. annuum* and *C. eximium* species, miR10 showed differences in only *C. eximium*, and miR23 showed a different expression trend in *C. annuum.* To validate the expression profiles of protein-coding genes targeted by miRNAs, we used qRT-PCR for all *Capsicum* species at different stages. The expression profiles of auxin-induced protein, class III HD-Zip protein, *TCP* TF, and serine carboxypeptidase protein, regulated by miR162, 166a, 319, and 016, respectively, showed opposite expression profiles from their target genes, as expected for miRNA targets (Figure 8). As expected, the expression of the remaining predicted targets was not opposite to their miRNA profiles in at least one species, which suggests that the activity of these genes could be determined by translational repression and/or by multiple miRNAs.

## 3. Discussion

Small RNAs, including miRNAs, are key regulators of biological processes such as biotic and abiotic stress tolerance, plant growth and development, metabolic pathways, and morphogenesis [28,29]. In plants, miRNAs regulate gene expression mainly by targeting mRNAs for cleavage and/or by translation inhibition of the target mRNAs during or after transcription [30]. Thus, miRNAs usually negatively regulate the accumulation of mRNAs and show an inverse correlated expression pattern in the same plant cells [31]. Such changes in the expression profile of plant genes play an important role in establishing specific phenotypes between plant species [32]. However, the molecular mechanisms underlying these changes are largely unknown. 

Although miRNAs have been identified in pepper fruits by using small RNA-seq [22,23], we lack a systemic comparison study of miRNA expression in pepper between domesticated and wild species, despite the worldwide importance of these fruits. In this study, we investigated the contribution of gene expression regulation by miRNAs in cultivated (*C. baccatum* and *C. annuum*) and wild (*C. chacoense* and *C. eximium*) *Capsicum* species at flower and two fruit development stages (6- and 25-dpa fruits, namely small and medium fruit stages). Our study identified 22 known and 27 novel miRNAs differentially expressed across *Capsicum* species that may be closely involved in biological processes controlling flower and fruit ripening, including fruit development, morphogenesis, pigmentation, and quality in wild and cultivated pepper species. 

### 3.1. Role of miRNAs and Their Regulators in Flowering across Capsicum Species

Flowering is an important biological process for plants that represents the phase transition from vegetative growth to reproductive growth, ensuring success in plant reproduction [33,34]. Several miRNAs have been reported to be a main regulator of the floral phase transition in many plants, including Arabidopsis [35], tomato [36], and apple [37]. For instance, miR156/157 and 172 are the two main key members of the aging pathway regulating *SPL* and *AP2-like* TFs [34]. These miRNAs play antagonistic but related roles in Arabidopsis flower induction, whereas a high level of miR156 extends the juvenile phase and delays flowering, and miR172 accumulation leads to early flowering [38,39]. As shown in Figure 4a, miR172 was highly accumulated in flowers in domesticated species, especially *C. annuum*, so cultivated species may exhibit early flowering as compared with wild species, which could be beneficial for pepper productivity. Similar to miR172, miR319 regulating *TCP* TFs showed high expression in *C. annuum*. Although *TCP* TFs have been more associated with leaf development and petal growth [40], recently Li et al. [41] described the function of these TFs as key regulators of flower development. Likewise, Wang et al. [42] reported that overexpression of *TCP8* significantly delayed flowering in Arabidopsis under long- and short-day conditions; additionally, high expression of *TCP4* promoted pistil abortion in *Prunus mume* [43]. Our results suggest that overexpression of miR319 in *C. annuum* negatively regulates *TCP* TF expression, which results in early flowering in this cultivated species and needs further confirmation.

Besides miR156/157, 172, and 319, many other miRNA families involved in the control of plant flowering time include miR159, 165/166, 167, 169, 171, 319, 390, and 399 [44]. miR159 regulates *GAMYB* TFs involved in the gibberellic acid (GA) pathway in different land plants as well as meristem formation and seed development [45,46]. Our results revealed a higher expression of miR159 than other miRNAs across species and stages; however, specifically at flowering stage, all species with the exception of *C. chacoense* showed similar expression. These results may suggest that GA content is an important player in flower initiation and development across *Capsicum* species. Furthermore, miR166b and 390 showed high expression in *C. annuum* and *C. baccatum*, respectively, and regulated LRR receptor-like serine/threonine-protein kinase (*LRR-RLK*). Previous studies of Arabidopsis reported the role of these proteins in floral organ abscission [47], in the main abscisic acid-mediated (ABA) signaling pathway and in early ABA perception [48]. Thus, miR166b and 390 may participate as negative regulators of flower organ development through the ABA pathway in cultivated species. 

Along with the known miRNAs mentioned above, novel miRNAs were upregulated during flower stage, mainly in wild species. For instance, miR17, 22, 23, and 24 showed high expression in *C. eximium*, whereas miR25 was highly expressed in *C. chacoense*. These miRNAs regulate genes coding for disease resistance proteins, suppressor of gene silencing 3-like (*SGS3*) proteins, and nonsense-mediated mRNA decay proteins, involved in plant pathogen defense and destruction of aberrant mRNAs [49,50]. The novel miRNAs we identified may be linked to plant pathogen protection against virus infection during flowering in wild *Capsicum* species that represent valuable germplasm resources for crop improvement.

### 3.2. Role of miRNAs and Their Regulators in Pepper Fruit Development

*Capsicum* species are highly diverse, and fruit attributes are one of the principal phenotypic differences among accessions [51]. Domestication events and continuous selection have increased the variability of fruit features such as shape, size, color, and aroma. Domesticated fruit-bearing crop species have largely increased their fruit size compared with those normally found in progenitor wild species [52,53]. As shown in Figure 1, domesticated species used in this study had large fruits, *C. annuum* having the largest. However, wild species had smaller fruits, with a round and elongated fruit shape for *C. chacoense* and *C. eximium*, respectively. miRNAs orchestrate different fruit development process such as fruit set, formation, shape, size, ripening, and quality in multiple plants including tomato [36,54,55], rice [56], cassava [57], *Lycium barbarum* [58], sweet potato [59], orange [60], blueberry [61], and diverse cucurbit species [62]. 

The hierarchical clustering in Figure 4 and Figure 5 for known and novel miRNAs, respectively, revealed that domesticated species were grouped, sharing similar miRNA expression patterns at fruit stages; however, some miRNAs showed high expression in *C. baccatum* versus other *Capsicum* species (Figure 5c,d). In addition to their role in plant vegetative versus reproductive phase change, miR156 and 172 have also been associated with fruit size in different species depending on the fruit type [63,64]. For instance, overexpression of miR156a reduced the fruit size and yield in tomato [65], whereas overexpression of miR172 in apple led to reduced fruit size and weight [66]. In our study, miR172 was uniquely expressed in *C. baccatum* at small fruit stage, whereas miR156 was highly abundant in *C. baccatum* at both fruit stages. Likewise, miR159 displayed different levels of upregulation in *C. baccatum* at both fruit stages. Overexpression of miR159 has been associated with abnormal ovule development that affects the initial fruit set, precocious fruit initiation, and seedless fruits in tomato [67]; in contrast, in strawberry, miR159 repression inhibited receptacle ripening and color formation [68]. The increased expression of miR156 and 159 in *C. baccatum* may interfere somehow in the fruit set and size of this species, which remains to be addressed. 

Furthermore, Nimmakayala et al. [69] reported an *Ankyrin* protein with acyltransferase activity associated with fruit weight in *C. annuum*. Similarly, Qin et al. [20] reported an acyltransferase involved in the selective sweep signals in the cultivated C. *annuum L. (Zunla-1)* versus its wild progenitor Chiltepin (*C. annuum* var. glabriusculum). Our results showed that miR12 with high expression in *C. baccatum* at middle fruit stage was related to regulation of an acyltransferase protein, which may be involved in the differences in fruit size between cultivated and wild pepper species. As well, we identified a *CLAVATA1* gene regulated by miR408. It has been reported in tomato that a mutation in the *CLAVATA3* gene involved in the CLAVATA-WUSCHEL pathway promoted stem-proliferation, increasing the meristem size, and resulting in the development of extra organs in flowers and larger fruits [70,71]. These results suggest that *CLAVATA* genes are implicated in pepper fruit size and are regulated by novel miRNAs. Moreover, Qin et al. [20] also reported an *ABC* transporter and Pleiotropic drug resistance protein 1 (*PDR1*) genes likely associated with the morphological and physiological differences between wild and domesticated pepper species. Likewise, it has been reported that the *ABC* transporter is a candidate gene at the *fw3.2* locus associated with fruit weight in tomato [72]. In pepper, *ABC* transporter has also been associated with fruit weight [69] and capsaicinoid content [73]. In our study, we identified two novel miRNAs, miR20 and miR21, regulating an *ABC* transporter and *PDR4* proteins, suggesting their importance for differences in pepper fruit development between wild and domesticated species.

In addition to their involvement in fruit size, miRNAs are also important for their involvement in the auxin signaling pathway regulating auxin responsive factors (*ARFs*) [74]. Auxin is a key hormone implicated in most plant organ developmental processes [75] and has been reported to affect fruit shape and development [32]. miR160 regulates *ARFs* in different plants such as Arabidopsis [76], cotton [77], and tomato [78]; however, in our study, miR162 was also found to regulate an auxin-induced protein. miR160 was downregulated across *Capsicum* species and fruit stages, whereas miR162 showed high expression at middle fruit stage in *C. baccatum*. Previous studies have reported that upregulation of miR160 targeting *ARF10* affects fruit shape in tomato [79], whereas downregulation alters the abscission of petal and anther, having an effect on tomato fruit set [78]. Together, miR160 and 162 may play an important role in the auxin signaling pathway in *Capsicum* species and thus are involved in regulating fruit shape and development. 

Quality and flavor are two important attributes of pepper fruits. Both features are strongly related to sugar and acid composition [23]. KEGG pathway analysis revealed that some of the miRNAs regulate major candidate genes involved in amino sugar and nucleotide sugar metabolism, starch and sucrose metabolism, and fructose-mannose metabolism pathways. Carbohydrate metabolism-related genes such as cellulose synthase, *FGGY* carbohydrate kinase, SlArf/Xyl2 and fructokinase proteins were regulated by miR10, 11, 13, and 15, respectively, which suggests that these novel miRNAs may be involved in modulating pepper fruit quality. Particularly, miR13 and 15 were downregulated in all species, whereas miR10 and 11 showed high expression at both fruit stages in *C. baccatum*. Likewise, novel miR06, which targets a methylenetetrahydrofolate reductase and is involved in folate metabolism, was upregulated in *C. baccatum* at both fruit stages, so novel miRNAs may also be involved in the concentration of the B vitamin group across *Capsicum* species. Furthermore, the novel miRNAs miR23 and 24 regulated a gene encoding 1-aminocyclopropane-1-carboxylate oxidase enzyme (*ACO*), which is directly involved in ethylene biosynthesis [80]. *ACO* is the rate-limiting step in ethylene production during certain dedicated processes such as fruit ripening in tomato [81,82]. *ACO* is regulated by the known miR396 in citrus, which inhibits ethylene biosynthesis [83]. Although pepper fruit ripening is widely classified as non-climacteric, patterns of ethylene production and respiratory rates vary across pepper species due to their wide diversity [84,85]. For instance, hot pepper (*C. frutescens*) fruit ripening is regulated by ethylene and ABA biosynthesis [86]. In addition, some varieties such as ‘Camelot’, ‘King Arthur’, and ‘Tabasco’ exhibit characteristics intermediate between climacteric and non-climacteric fruit ripening [87]. Another miRNA related to fruit ripening in tomato and *Lycium barbarum* involving the ethylene biosynthesis is miR164, regulating *NAC* TFs [58]. In tomato, *SlNAC4* RNAi-knockout plants showed delayed fruit ripening and a reduction of 30% of total carotenoid content, which suggests that *SlNAC4* is a positive regulator of ripening and carotenoid accumulation [88]. In our study, miR164 showed high accumulation at middle fruit stage in *C. chacoense*, which suggests its role as a negative regulator of fruit ripening in this species. Moreover, fruit ripening and softness are influenced by alpha-N-acetylglucosaminidase and hydrolase (hydrolyzing O-glycosyl compounds) [89,90], which are regulated by miRNAs such as the identified miR168 and 6478. 

Narrow genetic diversity which typifies cultivars of domesticated crops has increased the susceptibility of domesticated plants to major diseases [91]. Wild relatives of cultivated crops are recognized as a rich source of genes for disease resistance and stress tolerance. Our results showed different known and novel miRNAs regulating disease resistant genes. For instance, miRNAs such as miR165 and 398 were upregulated in the wild pepper relative *C. eximium*; these miRNAs were found to target aspartic proteinases *CDR1* (constitutive disease resistance 1). Along with their roles in plant development, aspartic proteinases have been associated with immune defense in pepper plants [92]. Additionally, we identified miR394 and miR403 that regulate an F-box protein. F-box genes are well known to be involved in hormone signaling and in response to abiotic stress in pepper [93]. Lim et al. [94] reported a novel F-box protein, *CaDIF1* (*C. annuum* Drought-Induced F-box Protein 1) in pepper. *CaDIF1*-silenced pepper plants exhibited a drought-sensitive phenotype, whereas *CaDIF1*-overexpressing plants exhibited ABA-sensitive and drought-tolerant phenotypes. MiR394 and miR403 were upregulated at both fruit stages in *C. baccatum*, which may suggest a lower accumulation of F-box proteins in this domesticated pepper species and therefore a high susceptibility of *C. baccatum* to drought stress. In addition, we identified miR403 which regulates an Argonaute (*AGO2*) protein. *AGO* proteins bind to small-interfering (si)RNAs and micro (mi)RNAs to target RNA silencing against viruses [95], suggesting that miR403 is directly involved in regulation of pepper defense response to virus infection. Furthermore, miR390 and miR22 associated with a leucine-rich repeat receptor kinase (*LRR* receptor-like protein) and miR396 associated with a late blight resistance protein were identified. Qin et al. [20] reported 10 disease resistance genes such as LRR and late blight resistance proteins showing a strong selective sweep signals in the cultivated peppers, indicating that these genes seemed to have been affected by selection during domestication. Our results support the involvement of miR390, miR396, and miR22 in pepper domestication. Overall, our results suggest that miRNAs identified, and their target genes, likely serve crucial regulatory roles in pepper related to vegetative to reproductive phase changes, fruit development and quality, and disease resistance across domesticated and wild pepper species. Further research to characterize the microRNAome in *Capsicum* will be required to confirm these inferences. 

## 4. Materials and Methods

### 4.1. Plant Materials

*C. baccatum* cv. Lemon drop, *C. annuum* cv. Cayenne, *C. chacoense* cv. PI 669106, and *C. eximium* cv. PI 645681 were grown in the greenhouse at West Virginia State University with the following conditions: 16 h of light at 26 °C and 8 h of darkness at 20 °C, relative humidity of 75 ± 2%, and plants were watered daily using manual irrigation. Flowers were uniformly sampled for unopened and fully matured buds from all cultivars, while fruit samples were collected at 6 and 25 days post-anthesis (dpa) from all four cultivars. All samples were harvested in three biological replications and frozen immediately in liquid nitrogen and stored at −80 °C for RNA isolation. 

### 4.2. Construction and Sequencing of Small RNA Libraries

Total RNA was isolated by using TRIzol reagent (Life Technologies, Carlsbad, CA, USA) according to the manufacturer’s instructions. RNA purity was achieved by using the RNA clean and concentrator kit (Zymo Research, Irvine, CA, USA) along with on-column DNaseI digestion to remove genomic DNA. Total RNA quality and quantity were measured by using the Agilent 2100 Bioanalyzer and Qubit 4 Fluorometer (Invitrogen, Carlsbad, CA, USA), respectively. Total RNA from three biological replicates was pooled for each sample before small RNA-Seq library preparation. At least 1.5 µg high-quality total RNA was used to construct each small RNA library and for sequencing by the Beijing Genomics Institute (Hong Kong) with the Illumina HiSeq 2500 platform (Illumina, San Diego, CA, USA). The small RNA-seq dataset was deposited in the NCBI SRA database under the accession number PRJNA718887. 

### 4.3. Bioinformatics Analysis of miRNAs

After sequencing, the quality of raw reads was ascertained by checking the adapter, GC distribution, average base content, and quality score of the distribution by using the fastqc program. The Cutadapt toolkit was used for removal of poor-quality reads, sequences with a poly-A tail, low-quality reads with ambiguous bases (“N”), reads with <18 or >30 bases, and adapter sequences [96]. Furthermore, the clean reads were BLAST-searched against the Rfam database (v14.1) [97] and Repbase [98] to identify and exclude known noncoding RNAs including rRNAs, scRNA, snoRNAs, snRNAs, and tRNAs. The remaining clean reads were searched against the miRBase database (v22.1, http://www.mirbase.org/, accessed on 1 August 2020) to identify known putative miRNAs [17]. The final miRNAs dataset underwent sequence length distribution and nucleotide preference analysis at each position. Meanwhile, the remaining unannotated sRNA sequences that were not mapped to any pre-miRNAs in miRbase were analyzed by using miRDeep2 [99] to predict potential novel miRNAs using the *C. annuum* cv. CM334 reference genome [19]. The prediction of novel miRNAs was based on previously reported criteria including (a) no more than four mismatches between the small RNA and the target (G-U bases count as 0.5 mismatches), (b) no more than two adjacent mismatches in the miRNA/target duplex, (c) no adjacent mismatches in positions 2 to 12 of the miRNA/target duplex (5′ of miRNAs), (d) no mismatches in positions 10 to 11 of the miRNA/target duplex, (e) no more than 2.5 mismatches in positions 1 to 12 of the miRNA/target duplex (5′ of miRNAs), and (f) a minimum free energy (MFE) of the miRNA/target > 75% [100,101]. Additionally, the putative precursor sequence was folded for each candidate novel miRNA by using RNAfold from the Vienna RNA software package [102]. Similarly, as for known miRNAs, for all the predicted novel miRNAs, properties including miRNA count, length, and nucleotide bias at each position were determined. 

### 4.4. Analysis of Differentially Expressed miRNAs

We used the R package DEGseq [103] to identify differentially expressed known and novel miRNAs across species and stages. The fold changes of miRNA expression were normalized by transcripts per million according to the formula: Normalized expression (NE) = Actual miRNA reads count/Total count of clean reads × 1,000,000 (Zhou et al., 2010).

### 4.5. Prediction of miRNA Targets and Enrichment Analyses 

The putative target genes of identified miRNAs were predicted by using psRNATarget [104] with a pepper transcriptome dataset [20]. Genes with a final sequence score ≤ 5 were considered potential candidate miRNA targets. The target genes were functionally annotated by using the Kyoto Encyclopedia of Genes and Genomes (KEGG, http://www.genome.jp/kegg/, accessed on 1 September 2020) pathway database with the BlastKOALA sequence similarity tool [105]. Pathways with false discovery rate (FDR) ≤ 0.5 were considered significantly enriched. Gene Ontology (GO) analysis of these genes involved using Blast2GO with a cutoff E-value of 10^−5^ (http://www.blast2go.com, accessed on 4 May 2021) [106]. The Reads Per Kilobase of transcript expression values from leaf, stem, and placenta tissues (6, 16, 25 dpa) for the identified target genes were retrieved from published RNA-seq data [19] and used to generate a heatmap with the ClustVis web tool (https://biit.cs.ut.ee/clustvis/, accessed on 1 September 2020). 

### 4.6. Quantitative RT-qPCR and Stem-Loop RT-qPCR

To validate our results from the bioinformatics-based analysis, we used stem-loop RT-qPCR for miRNAs and RT-qPCR for target genes in flower, small fruit (6-dpa) and medium fruit (25-dpa) tissues with three biological replications across the *Capsicum* species analyzed. The RT-qPCR primers were designed from miRNA sequences as described [107] (Table S5). Total RNA used for small RNA sequencing was used for reverse transcription using Superscript IV reverse transcriptase. RT-qPCR analysis was achieved with the Universal SYBR Green Master Mix (Thermo Fisher Scientific, USA) on the StepOne Plus Real-Time PCR System (Applied Biosystems, USA) with the following conditions: 95 °C for 10 min, followed by 40 cycles of 95 °C for 15 s, 56 °C for 30s, and 72 °C for 15 s, and melting curve analysis from 65 to 95 °C. To normalize gene expression, small nuclear RNA (snRNA) U6 and β-tubulin were internal controls for miRNA and target genes, respectively. All RT-qPCR reactions involved three technical replicates, and the relative gene expression of miRNAs and targets was estimated by the 2^-ΔΔCt^ method [108]. The oligonucleotide primers corresponding to the predicted target genes are in Table S6.

## 5. Conclusions

Considering the global importance for pepper worldwide, we used high-throughput sequencing and identified 22 known miRNAs and 27 novel miRNAs differentially expressed in flower and pepper fruits at different developmental stages across 4 *Capsicum* species. Analysis of differential expression patterns combined with target prediction suggested key roles for these miRNAs in controlling flower time and pepper fruit development in cultivated and wild species. The results expand the study of miRNAs in plants by providing a better understanding of their essential roles in miRNA-based regulation processes in pepper. Similarly, our results provide insight into the biology, evolution, and domestication process of *Capsicum* species, accelerating the agricultural applications of the miRNAs, the genes of their biogenesis pathway and providing targets for future investigation in pepper and other plants.

## Figures and Tables

**Table 1:** Expression level based on transcripts per million (TPM) of known miRNAs identified in *Capsicum* species at flowering, small fruit, and middle fruit stages.

	*Capsicum baccatum*			*Capsicum annuum*			*Capsicum chacoense*			*Capsicum eximium*			
*Name*	Flowering	Small Fruit	Middle Fruit	Flowering	Small Fruit	Middle Fruit	Flowering	Small Fruit	Middle Fruit	Flowering	Small Fruit	Middle Fruit	*miRNA Sequence*
*miR156*	20	10	25	10	0	6	6	5	0	9	7	9	*UGACAGAAGAGAGUGAGCAC*
*miR157*	47	12	0	281	0	0	0	0	68	132	0	9	*UUGACAGAAGAUAGAGAGCAC*
*miR159*	7417	32107	22478	10667	3720	2206	578	1893	1419	6182	3176	3912	*UUUGGAUUGAAGGGAGCUCUA*
*miR160*	0	36	30	31	0	0	0	0	19	14	0	0	*UGCCUGGCUCCCUGUAUGCCA*
*miR162*	26	733	1252	385	260	262	6	30	19	187	7	45	*UCGAUAAACCUCUGCAUCCAG*
*miR164*	194	55	598	377	52	334	25	123	1422	83	86	204	*UGGAGAAGCAGGGCACGUGC*
*miR165*	31	17	14	7	0	0	0	0	6	0	0	35	*GUUGAGGGGAAUGUUGUCUGG*
*miR166a*	1699	7225	7342	2025	729	815	383	1498	1167	1234	1523	1477	*UCGGACCAGGCUUCAUUCCCC*
*miR166*	0	16	25	1718	313	0	29	183	24	713	624	631	*GGGGAAUGUUGUCUGGCUCG*
*miR167*	116	354	725	302	8	44	0	29	7	0	16	51	*UGAAGCUGCCAGCAUGAUCUA*
*miR168*	11	46	79	32	10	52	0	0	0	35	0	13	*CCCGCCUUGCAUCAACUGAAU*
*miR171*	77	363	495	372	102	42	0	38	6	106	26	41	*UAUUGGCCUGGUUCACUCAGA*
*miR172*	68	21	15	108	0	8	11	0	15	34	0	0	*AGAAUCUUGAUGAUGCUGCAU*
*miR319*	2098	403	121	4461	154	90	290	126	54	2027	604	484	*UUGGACUGAAGGGAGCUCCC*
*miR390*	320	305	197	92	8	0	0	0	0	45	0	0	*AAGCUCAGGAGGGAUAGCGC*
*miR394*	29	303	193	118	19	0	0	8	0	38	0	0	*UUGGCAUUCUGUCCACCUCC*
*miR395*	106	26	17	63	0	41	0	44	45	107	0	0	*CUGAAGUGUUUGGGGGAACUC*
*miR396*	144	2668	3232	778	507	1681	9	282	351	292	27	144	*UUCCACAGCUUUCUUGAACUG*
*miR398*	25	20	428	641	0	79	0	196	20	1618	0	3520	*UGUGUUCUCAGGUCACCCCUU*
*miR403*	123	1819	1584	35	235	235	0	55	53	254	14	86	*UUAGAUUCACGCACAAACUCG*
*miR408*	0	7	117	0	0	38	0	0	8	110	147	79	*UGCACUGCCUCUUCCCUGGCU*
*miR6478*	2750	15436	2122	10213	6236	4524	1001	3789	1508	9530	2404	4669	*CCGACCUUAGCUCAGUUGGUAGA*

**Table 2:** Expression level based on transcripts per million (TPM) of novel miRNAs identified in *Capsicum* species at flowering, small fruit, and middle fruit stages.

		*Capsicum baccatum*			*Capsicum annuum*			*Capsicum chacoense*			*Capsicum eximium*			
*Name*	MFE	Flowering	Small Fruit	Middle Fruit	Flowering	Small Fruit	Middle Fruit	Flowering	Small Fruit	Middle Fruit	Flowering	Small Fruit	Middle Fruit	*miRNA Sequence*
*miR01*	−18.8	57	11265	465	9	0	0	0	11	0	231	236	89	*AACCCUGAACCCUGAACCCU*
*miR02*	−30.7	11	85	11380	0	0	15	0	0	0	0	61	253	*AGGGAUGGCCUUGGCUCAGC*
*miR03*	−49.9	158	120	1468	54	30	29	126	126	172	102	214	86	*GAAGUCCUCGUGUUGCAUCCCU*
*miR04*	−47.9	0	252	23	26	36	19	0	0	0	25	0	0	*GACUAGGACGGUCUGAGGCUU*
*miR05*	−46.3	1252	12908	32	640	568	432	92	214	60	402	137	130	*GCACCAGUGGUCUAGUGGUAGAAU*
*miR06*	−18.3	75	715	584	359	183	131	8	122	38	381	36	136	*GCCCGUCUAGCUCAGUUGGUAGA*
*miR07*	−62.6	7487	0	10012	2965	0	2156	4328	0	14605	6921	4728	5898	*GCCGGCCGGGGGACGGACUG*
*miR08*	−56.1	0	55	592	17	0	12	0	22	66	49	13	35	*GCCGUCUUAGCUCAGCGGUA*
*miR09*	−30.3	0	89	1532	68	90	48	0	0	320	165	0	0	*GCCGUCUUAGCUCAGUGGUAGAGC*
*miR10*	−30.8	865	3358	64	416	0	0	62	452	0	654	389	309	*GCGCCUGUAGCUCAGUGGAUA*
*miR11*	−44.8	0	42	1827	0	0	0	0	0	0	0	0	0	*GCGGAAGAUCCUGAAUUUGAGACU*
*miR12*	−23.7	239	530	8656	194	215	173	268	148	0	274	217	150	*GCGGGGAUAGCUCAGUUGGGAGA*
*miR13*	−27.6	7777	18	140	6089	4959	2735	4746	9380	705	7712	8554	8715	*GCGUCUGUAGUCCAACGGUUAGG*
*miR14*	−26.5	1317	8787	118	7068	18987	16637	1832	4800	9219	11268	421	2526	*GCUCAGUGGUAGAGCAUUUGACU*
*miR15*	−37	1854	2927	265	1155	549	314	1232	2325	2893	2456	1162	1624	*GGAUGCGAUCAUACCAGCACU*
*miR16*	−34.3	697	543	3629	224	125	0	754	750	550	340	742	371	*GGGAAGUCCUCGUGUUGCAUCCCU*
*miR17*	−29.7	539	468	21	609	131	148	121	383	302	753	128	288	*GGGAUUGUAGUUCAAUCGGUCAGA*
*miR18*	−15.9	0	0	4668	0	493	772	0	0	1690	0	0	0	*GGGGAUGUAGCUCAAAUGGU*
*miR19*	−18.1	8955	3485	0	1592	0	0	7949	8286	0	3517	10077	7444	*GGGGAUGUAGCUCAAAUGGUAGA*
*miR20*	−23	0	0	0	0	1815	1882	0	0	6380	5022	0	0	*GGGGAUGUAGCUCAGAUGGUA*
*miR21*	−24.9	11206	4473	0	3305	0	0	14304	13268	0	0	16839	12566	*GGGGAUGUAGCUCAGAUGGUAGA*
*miR22*	−55.1	210	501	456	455	312	0	190	321	413	1032	118	372	*GUCGAUAUGUCCGAGUGGUUAAGG*
*miR23*	−50.5	1781	2755	12	1676	811	726	771	1077	1094	2750	1114	1937	*GUGGACGUGCCGGAGUGGUUAUC*
*miR24*	−40.6	50	0	0	56	11	0	31	33	32	89	68	60	*GUGGGCGUGCCGGAGUGGUUAUC*
*miR25*	−36.6	0	0	0	0	0	0	285	103	115	0	143	0	*UAGUGGUAUGAUUCUCGCUU*
*miR26*	−37.8	98	0	0	0	238	112	438	235	0	429	668	159	*UAGUGGUCAGGACAUUGGACU*
*miR27*	−30	0	270	62	33	0	0	0	0	0	20	0	0	*UCACCAUCUUUCGGCUGAGAUU*

## Data Availability

The small RNA-seq dataset was deposited in the NCBI SRA database under the accession number PRJNA718887 https://www.ncbi.nlm.nih.gov/sra/PRJNA718887, accessed on 4 May 2021.

## Supplementary Materials

The following are available online at https://www.mdpi.com/article/10.3390/ijms22094866/s1.
